# Commissioning and clinical implementation of the first commercial independent Monte Carlo 3D dose calculation to replace CyberKnife M6™ patient‐specific QA measurements

**DOI:** 10.1002/acm2.13046

**Published:** 2020-10-25

**Authors:** Maaike T. W. Milder, Markus Alber, Matthias Söhn, Mischa S. Hoogeman

**Affiliations:** ^1^ Department of Radiotherapy Erasmus MC University Medical Center Rotterdam Rotterdam Netherlands; ^2^ Section for Medical Physics Department of Radiation Oncology University Clinic Heidelberg Heidelberg Germany; ^3^ Scientific RT GmbH Munich Germany

**Keywords:** independent dose calculation, patient‐specific QA, pre‐treatment QA

## Abstract

**Purpose:**

To report on the commissioning and clinical validation of the first commercially available independent Monte Carlo (MC) three‐dimensional (3D) dose calculation for CyberKnife robotic radiosurgery system® (Accuray, Sunnyvale, CA).

**Methods:**

The independent dose calculation (IDC) by SciMoCa® (Scientific RT, Munich, Germany) was validated based on water measurements of output factors and dose profiles (unshielded diode, field‐size dependent corrections). A set of 84 patient‐specific quality assurance (QA) measurements for multi‐leaf collimator (MLC) plans, using an Octavius two‐dimensional SRS1000 array (PTW, Freiburg, Germany), was compared to results of respective calculations. Statistical process control (SPC) was used to detect plans outside action levels.

**Results:**

Of all output factors for the three collimator systems of the CyberKnife, 99% agreed within 2% and 81% within 1%, with a maximum deviation of 3.2% for a 5‐mm fixed cone. The profiles were compared using a one‐dimensional gamma evaluation with 2% dose difference and 0.5 mm distance‐to‐agreement (Γ(2,0.5)). The off‐centre ratios showed an average pass rate >99% (92–100%). The agreement of the depth dose profiles depended on field size, with lowest pass rates for the smallest MLC field sizes. The average depth dose pass rate was 88% (35–99%). The IDCs showed a Γ(2,1) pass rate of 98%. Statistical process control detected six plans outside tolerance levels in the measurements, all of which could be attributed the measurement setup. Independent dose calculations showed problems in five plans, all due to differences in the algorithm between TPS and IDC. Based on these results changes were made in the class solution for treatment plans.

**Conclusion:**

The first commercially available MC 3D dose IDC was successfully commissioned and validated for the CyberKnife and replaced all routine patient‐specific QA measurements in our clinic.

## 1 INTRODUCTION

To ensure safe dose delivery in stereotactic radiotherapy, uncertainties and errors in dose delivery must be minimized by an extended and strict quality assurance (QA) protocol. An established part of a QA protocol is the patient‐specific pre‐treatment verification of the calculated dose, either by measurements or by checking the monitor units with an independent dose calculation (IDC).^1^

Patient‐specific QA measurements can be performed using chambers, film, or diode arrays.^2^, ^3^, ^4^, ^5^, ^6^, ^7^ Especially for stereotactic CyberKnife treatment plans, both measurement equipment and analysis require stringent quality criteria. Internationally acknowledged gamma criteria of 2% dose difference and 2 mm distance‐to‐agreement Γ(2,2) are insufficient to detect possible errors relevant during CyberKnife dose delivery.^2^, ^4^, ^8^ However, measurements are costly by consuming valuable personnel and machine time. Besides this, despite fulfilling the strict criteria, the relevant errors that can be picked up are limited and mainly refer to problems regarding the delivery system.^9^, ^10^, ^11^ It is more efficient that these types of issues are addressed by proper commissioning and machine QA.^8^

An alternative to pre‐treatment measurements is an IDC.^1^, ^8^ This method recalculates the dose independent of a vendor treatment planning system (TPS), based on the treatment plan parameters for the given plan, and can range between a point dose calculation to a full three‐dimensional (3D) Monte Carlo calculation.^12^, ^13^, ^14^ Independent dose calculation platforms have been developed for the CyberKnife fixed cone and Iris™ collimators^15^, ^16^ and recently also for the newly developed MLC collimator.^17^, ^18^ None of these solutions offers a (MC) 3D IDC that is commercially available.

This paper describes the commissioning and clinical implementation of the first commercially available 3D Monte Carlo dose engine (SciMoCa RT, Munich) for two CyberKnife® M6™ robotic radiosurgery system (Accuray Inc., Sunnyvale, CA) in order to replace patient‐specific QA measurements. To this end, the beam model provided by SciMoCa was compared to water tank reference measurements for all three collimator sets. Using SPC, treatment plans outside tolerances were detected in array measurements and IDCs and were further analysed. As the vast majority of our patient cohort is treated using the MLC collimator, retrospectively a set of 84 patient MLC plans was used for this evaluation.

## 2 MATERIALS AND METHODS

### 2.A Modeling of the CyberKnife accelerators

The SciMoCa algorithm has been described in Ref. [26] for general purpose linear accelerators with MLC. The CyberKnife implementation uses the same patient/phantom transport code, but differs in source and collimator models. The latter are purpose‐built for fixed cones and Iris, whereas the MLC model was adapted from the model described in Ref. [26] with respect to transmission, leakage, and additional fixed collimation elements in the assembly. The source model is comprised of four virtual sources (primary, primary collimator and other head scatter, beam filter scatter, contamination electrons), whereby the scatter components amount to only about 3.3% of total energy fluence at the maximum field size of the MLC, and about 1.7% for the 60 mm cone.

The three beam models (one for each collimator type) of a CyberKnife M6 share the same source model, which was derived from water phantom depth dose curve (DDC) and output factor (OF) measurements obtained with the Incise2 MLC. Input for the models was identical to the data obtained during CyberKnife commissioning: a DDC, a set of off center ratios (depth 15, 50, 100, 200, and 300 mm) and an OF for each field size summarized in Table 1. For the MLC cross profiles were included. Reference depth for all collimators and field sizes was 1.5 cm.

**Table 1:** CyberKnife field sizes. X‐axis in leaf travel direction, Y‐axis perpendicular to it.

Field size												
Fixed/Iris (mm)	5	7.5	10	12.5	15	20	25	30	35	40	50	60
MLC X (mm)	7.6	15.4	23.0	30.8	38.4	46.2	53.8	69.2	84.6	100.0	115.0	
MLC Y (mm)	7.7	15.4	23.1	30.8	38.4	46.2	53.9	69.3	84.7	100.1	100.1	

Input DDCs and OFs for the fixed cones and Iris fields were used for validation. All measurements were performed with a PTW 60012 unshielded diode in a PTW MP3 tank. Measurements were corrected for small field detector response using correction factors as published by Francescon et al.^19^, ^20^ Diameters of circular cones were calibrated from cross‐profiles to account for manufacturing tolerances in the order of 0.05 mm. The maximum energy of both machines differed by 100 keV and the electron spot radius by 0.08 mm.

### 2.B Commissioning

#### 2.B.1 Beam model validation

To validate the six individual beam models of the two CyberKnife systems, calculated OFs, DDCs, and off‐center ratios (OCRs) were compared to corresponding measurements for a range of field sizes (Table 1). OFs were calculated using a voxel size of 1 × 1 × 1 mm^3^ for fields ≤ 35 mm (fixed and Iris) or 30.8 × 30.8 mm^2^ (MLC) and 1.8 × 1 × 1.8 mm^3^ for larger fields with a statistical uncertainty of 0.1%. The statistical uncertainty is defined as the mean uncertainty of all voxels with dose >70% of the max dose, computed from 64 batches simulated with different random seeds. Depth dose curves were calculated with a voxel size of 1 × 1 × 1 mm^3^ for fields ≤ 30.8 × 30.8 mm^2^ and 1.8 × 1 × 1 mm^3^ for larger fields. OCRs were calculated with a voxel size of 0.5 × 1 × 0.5 mm^3^ for fields ≤ 15.4 × 15.4 mm^2^ and 1 × 2 × 1 mm^3^ for larger fields. These calculations had 0.25% statistical uncertainty. Comparisons between calculations and measurements were based on a 1D gamma evaluation, using Γ(1,0.5) and Γ(2,0.5), respectively.^21^ Analysis included the build‐up region.

#### 2.B.2 Independent dose calculation of clinical plans using SciMoCa

SciMoCa was used to retrospectively recalculate a set of 84 patient plans (35 and 49 on CyberKnife 1 and 2), using 1.5 mm^3^ resolution and 0.5% statistical uncertainty. The two systems are dosimetrically similar, but require different beam models in the TPS. In practice these models produce identical plans. All plans were hypofractionated and generated using the MLC in combination with the finite size pencil beam (FSPB) algorithm, the only available at the time, using the voxel size of the planning CT (resolution in plane 1 × 1 mm, slice thickness depending on the treatment site 1–2 mm). Treatment sites ranged from pancreas (21), liver (16), H&N (15) and prostate (4), to oligometastases (25) with a PTV size ranging between 30 and 300 cc. Clinical plans were generated using Multiplan® (version 5.1.3). Dose comparison was performed using an in‐house developed software platform for 3D gamma evaluation Γ(2,1), using global maximum, and 10% dose cutoff. The SciMoCa algorithm assigns material properties according to mass density, using ICRU tabulated values. For example, a voxel with density 1.45 g cm^−3^ would be interpreted as “ICRU bone with density 1.45,” and similar for lung for voxels with density between 0.75 and 0.011 g cm^−3^.

#### 2.B.3 Establishing action levels

For every new QA method, appropriate action levels need to be established to identify differences between dose calculated by the TPS and delivered dose. Action levels for IDCs were determined using statistical process control (SPC).^22^ This method has been described previously for radiotherapy quality assurance, such as linac QA,^23^ image‐guidance QA,^24^ IMRT dose verification^25^ and similar to our application, independent monitor unit calculation^26^ and the replacement of patient‐specific QA measurements by IDCs.^27^ Using SPC, chronological processes such as patient‐specific QA can be evaluated in control charts that show how the process randomly varies over time. Control charts typically show a central average line and statistically determined upper and lower control limits. In control charts, in contrast to the general conception in radiotherapy, systematic errors are defined as points outside the action levels. Besides systematic errors, the charts will show if the average or the random variation changes due to alterations in the treatment process. To set action levels in CyberKnife plan QA, two charts were used; an average chart, displaying the average and random spread of the measurements and a range chart that displays the difference between successive measurements and their average value. The range R was calculated according to Eq. (1).

(1) $$R_{i}=\left|x_{i}‐x_{i‐1}\right|$$

where x represents an individual, successive QA measurements. This leads to the following equations for the center lines and upper and lower thresholds in the average (A) and range (R) charts:

(2) $$A_{c}=\bar{x}$$

$$A_{u}=A_{c}+3\frac{\bar{R}}{d_{2}\sqrt{n}}=A_{c}+1.88\bar{R}$$

$$A_{l}=A_{c}‐3\frac{\bar{R}}{d_{2}\sqrt{n}}=A_{c}‐1.88\bar{R}$$

(3) $$R_{c}=\bar{R}$$

$$R_{u}=\left(1+3\frac{d_{3}}{d_{2}}\right)\bar{R}=3.267\bar{R}$$

$$R_{l}=\left(1‐3\frac{d_{3}}{d_{2}}\right)\bar{R}=‐1.267\bar{R}$$

The factors $d_{2}$ and $d_{3}$ are tabulated and are valid for a subgroup size of n=2 where each measurement can be treated as an independent data point.^23^, ^28^ In this case $R_{l}$ will be effectively set to 0.

### 2.C Comparison to pre‐treatment measurements

The intended use of the SciMoCa IDCs is to replace patient‐specific QA measurements. To ensure that this can be safely done, a risk assessment of the QA program, in line with AAPM TG100, needs to take place. As part of this, for the same set of 84 patients that received an IDC, individual pre‐treatment measurements were re‐evaluated using the SPC method. Identical gamma criteria as in IDCs were used: Γ(2,1), using the global maximum, and a 10% dose cutoff. Plans outside the action levels were further analyzed.

#### 2.C.1 Measurements of QA plans

The clinical Incise2 MLC‐based FSPB treatment plans of 84 patients, were matched with and recalculated on the CT scan of an Octavius 1000SRS (PTW, Germany) array embedded in a solid water slab phantom using the workflow offered by the Multiplan software. For all plans a two‐dimensional (2D) coronal dose plane was exported at the height in the phantom corresponding to the measurement plane in the array. Due to the angular dependence of the SRS1000 array, all beams were delivered with the linac head perpendicular to the phantom surface.^29^

This detector array consist of 977 MicroLion liquid‐filled ionization chambers. The spacing between the chambers in the high‐resolution area (5.5 × 5.5 cm^2^) is 2.5 mm. In the low resolution area, filling the remainder of the 11 × 11 cm^2^ area, the distance between the chambers is 5 mm. The use of the 1000SRS array for the CyberKnife has been investigated and found sufficiently accurate for patient‐specific QA measurements.^4^, ^6^ Our 1000SRS array has a fixed geometry with a slab phantom in which a set of three fiducial markers are embedded. Pre‐treatment alignment on the CyberKnife is based on a fiducial match between two stereoscopic x‐ray images with digitally reconstructed radiographs based on the treatment planning CT, ensuring optimal alignment between planned and delivered geometry.

A 2D gamma analysis was performed in Verisoft (v 7.0, PTW, Freiburg, Germany) for the two CyberKnife systems separately. No additional geometrical shift of the dose planes, to obtain optimal pass rates, was allowed in the analysis to prevent biased gamma results.

## 3 RESULTS

### 3.A Validation of the beam models

**Fig. 1:**
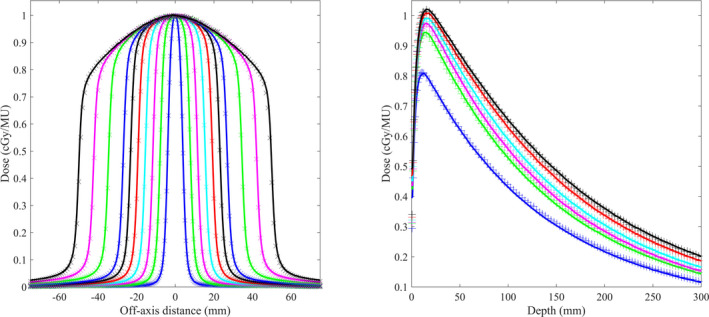
[Left] Measured (solid lines) and calculated (crosses) off‐axis beam profiles of increasing field sizes of the Incise2 collimator. [Right] Measured (solid lines) and calculated (crosses) depth dose curves of increasing field sizes of the Incise2 collimator. Field sizes: 7.6 × 7.7 mm^2^ (blue), 15.4 × 15.4 mm^2^ (green), 23.0 × 23.1 mm^2^ (magenta), 38.4 × 38.4 mm^2^ (cyan), 69.2 × 69.3 mm^2^ (red), 100 × 100.1 mm^2^ (black).

Absolute DDCs and OFs, and relative cross profiles calculated by SciMoCa were compared to measurements in a homogenous water phantom using a Γ(2,0.5) and Γ(1,0.5), respectively.^18^ Figure 1 shows measured versus calculated dose profiles (crossplane) and depth curves for increasing fields sizes of the Incise2 MLC.

Of the calculated OF for both CyberKnife systems 99% agreed within 2% and 81% within 1%, the maximum deviation of 3.2% is associated with a cone size of 5 mm (Table 2).

**Table 2:** Comparison of measured and calculated OF for two CyberKnife systems.

	CyberKnife 1			CyberKnife 2		
	Fixed	Iris	MLC	Fixed	Iris	MLC
Mean relative error (and range) %	0.32 (−1.25–1.10)	−0.21 (−1.58–0.55)	0.53 (0–1.20)	0.35 (−3.16–2.15)	‐0.12 (−1.41–0.33)	0.57 (−0.10–1.20)
Within 1%	9/12	10/12	10/11	7/12	11/12	10/11
Within 2%	12/12	12/12	11/11	10/12	12/12	11/11

Table 3 summarizes the comparison between the measurements and calculations. The off‐centre ratios showed an average pass rate >99% (92–100%) using Γ(1,0.5). The average depth dose pass rate was 88% (35–99%), where the agreement strongly depended on field size. The lowest pass rates were associated with the smallest MLC field sizes. DDCs were compared in terms of absolute dose. A difference in the OF propagates to a global difference in the DDC.

**Table 3:** Pass rates from the comparison between water tank validation measurements and calculations by SciMoCa for two CyberKnife systems.

	CyberKnife 1			CyberKnife 2		
	Fixed	Iris	MLC	Fixed	Iris	MLC
DTA = 0.5 DD ≤ 1%						
OCR	100	100	100 (98–100)	100	100	99 (92–100)
DDC	63 (10–97)	84 (43–93)	70 (5–99)	77 (35–91)	81 (58–91)	59 (3–93)
DTA = 0.5 DD ≤ 2%						
OCR	100	100	100 (99–100)	100	100	100 (98–100)
DDC	79 (35–99)	92 (87–93)	90 (36–99)	91 (85–92)	89 (75–93)	81 (25–93)

### 3.B Clinical validation

#### 3.B.1 Establishment of action levels

**Fig. 2:**
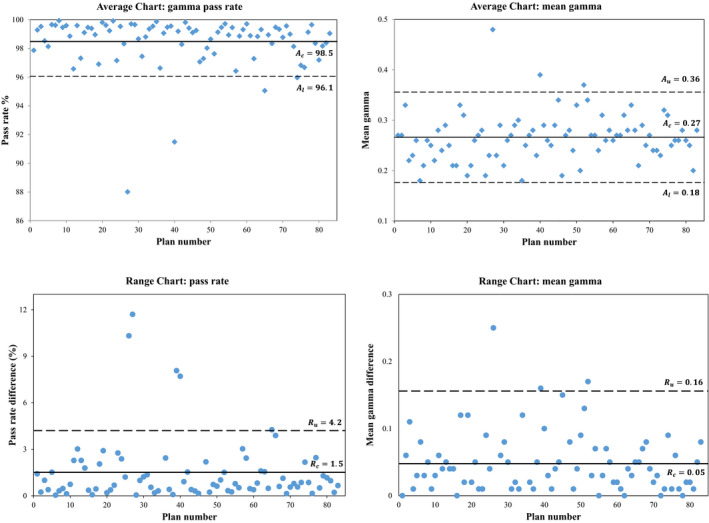
Ga*mma analysis of the comparison between IDCs and TPS. Average and range charts for gamma pass rate and mean gamma.*

Table 4 shows a summary of the SPC using average and Range charts that are displayed in Fig. 2. The mean pass rate of the measurements was 89% (range 49–100%) and 97% (range 89–100%) for the two CyberKnife systems. The mean pass rate of the IDCs was 98% (range 88–100%).

**Table 4:** Results of two‐dimensional (2D) gamma analysis of measured versus TPS dose on CyberKnife 1 and 2 and of the three‐dimensional (3D) gamma analysis of IDC vs TPS dose. Γ(2,1), dose cutoff at 10%. $R_{l‐pass}$ and $R_{l‐mean}$ are 0 by default.

	Measurement vs TPS — CyberKnife 1 (35 plans)	Measurement vs TPS — CyberKnife 2 (49 plans)	IDC vs TPS
**Average‐chart results**			
$A_{c‐pass}$(%)	89	97	98
$A_{u‐pass}$ ^a^	100	100	100
$A_{l‐pass}$(%)	69	93	96
Plans outside $A_{u‐pass}$ and $A_{l‐pass}$	3	3	4
$A_{c‐mean}$	0.55	0.46	0.27
$A_{u‐mean}$	0.89	0.59	0.36
$A_{l‐mean}$	0.21	0.33	0.18
Plans outside $A_{u‐mean}$ and $A_{l‐mean}$	2	0	3
**Range‐chart results**			
$R_{c‐pass}$(%)	10	2	2
$R_{c‐mean}$	0.18	0.07	0.05
$R_{u‐pass}$(%)	33	7	4
$R_{u‐mean}$	0.58	0.23	0.16
Number of plans outside $R_{u‐pass}$	1	0	5
Number of plans outside $R_{u‐mean}$	0	0	3
Total number of unique plans outside action levels	6 (3)	3 (3)	13 (5)

^a^ More than 100%.

#### 3.B.2 Plans exceeding the action levels

Table 4 and Fig. 2 show several plans that exceed the action levels: systematic errors. A single, unique patient plan could cause up to four systematic errors if it exceeds the control levels in all four control charts used in this analysis. In the measurements, six unique plans failed. These systematic errors could, after further analysis not be attributed to problems in treatment planning. In the IDCs five unique plans were outside the action levels. One plan exceeded the action levels in all four control charts. The systematic errors from the IDC have different origins, as detailed in the discussion.

#### 3.B.3 Comparison of IDCs and measurements

One plan exceeded the action levels in both the measurements and IDCs. However, none of the other systematic errors in the IDCs appeared in the analysis of the measurements and vice versa. The Pearson correlation coefficient between measurements and IDCs for the gamma pass rate and gamma mean ranges between −0.3 and 0.5.

## 4 Discussion

The first commercially available independent 3D MC IDC algorithm for all three collimator types of the CyberKnife was developed by Scientific RT on initiative of the Erasmus MC and validated together. The calculations showed excellent agreement with measurements in a homogeneous water phantom using very strict evaluation criteria of Γ(2,0.5). Including the build‐up region and all three collimators up to the smallest field size of 5 mm, 88% of the calculated and measured points passed Γ(2,0.5), excluding the smallest field sizes < 2 × 2 cm this value increased to 93%. The largest deviations in DDC occurred for the smallest MLC fields. Our results are comparable to previous work on in‐house developed IDCs.^15^, ^16^, ^17^, ^18^

The agreement in dose calculated by Multiplan and SciMoCa is very high. The average gamma pass rate Γ(2,1) 98% and mean 0.27 found in this work for 3D IDCs are well above the advised numbers of AAPM TG 135 of Γ(2,2) ≥ 90%.^8^ The pass rates agree well with earlier work on CyberKnife patient‐specific QA.^2^, ^15^, ^17^, ^18^ Previously, similarly high agreement between Acuros and SciMoCa calculated dose distributions had been reported.^30^

The pass rates of the pre‐treatment measurements show a strong learning curve associated with the in‐house development of a dedicated phantom. The gamma pass rate increased from 89%, for the initial set of measurements on CyberKnife system 1, to 97% for the subsequent set on system 2 coinciding with the change to the dedicated phantom. Similarly, the gamma mean decreased from 0.55 to 0.46. SPC is a valuable tool to visualize these trends in QA. The gamma pass rates in this study exceed those of similar work.^2^, ^3^, ^4^, ^6^ This can be attributed to the use of a dedicated CyberKnife QA phantom and the beam delivery perpendicular to the array surface, to avoid angular correction of the array response.

Action levels for gamma pass rate and gamma mean were set using SPC. Plans exceeding the action levels were further analyzed. In the measurements 5 out of 6 systematic errors could be explained by a scaling factor in the dose attributable to the initial absence of a dedicated phantom. The fourth case corresponds to a plan with a large target volume, due to which the high dose gradients overlapped with the low resolution region of the diode array. Hence, none of these systematic errors could be attributed to either machine or TPS quality issues. The origins of the five plans causing systematic errors in the IDCs are diverse. The target in the plan that exceeded the threshold in all four charts was located very close to the surface, in the buildup region. Larger deviations between algorithms can be expected for these type of plans. In clinical practice we have since seen more deviations in IDCs for superficial targets. A second plan outside tolerance showed a dose difference at the interface between soft tissue and bone. The clinical impact of this effect was limited as the dose constraints and the coverage were met using both algorithms. The remaining three cases were boost plans for the head and neck region. In general the presence of air close to the target is limited in these plans. In the three cases outside the tolerances, of sixteen head and neck cases analyzed in total, the unclipped PTV extended in air. Recalculation of these five plans with the MC algorithm, now available for MLC, improved the agreement in dose except for the first case. Based upon these results changes have been made in our class solutions.

The correlation between gamma parameters for patient‐specific QA measurements and IDCs was low with a Pearson correlation coefficient for the gamma pass rate and gamma mean ranging between −0.3 and 0.5. While the IDC was able to detect clinically relevant problems in the treatment plans, the measurements only revealed issues with the QA method. The fact that one plan causes a systematic error in both IDC and measurements seems a coincidence, as the origin of the deviation was different. Based on these results and an extensive period of testing the MLC,^31^ we have replaced our patient‐specific QA measurements by IDCs. Such a change in the QA program should be accompanied by a risk analysis in line with AAPM TG100. Issues that previously might have been intercepted only during patient‐specific QA measurements, must be picked up during machine QA. In our institute we work with class solutions for treatment sites. When introducing a new treatment site or plan technique a set of measurements is performed to validate the deliverability before relying on IDCs only. Plans outside protocol or class solution always receive a pre‐treatment measurement. End‐to‐end tests are performed when changes, such as a new TPS version, are introduced in the clinical practice. Upon replacement of the pre‐treatment measurements by IDCs a set of plans reflecting the clinical population was added to our QA program.

In this study, we focused on gamma parameters, however, the IDC method also allows us to look at clinically relevant differences in DVHs parameters. This is often used in our clinical practice and is a valuable additional tool to detect issues in treatment planning.^32^ Also, potential future development of log file analysis could further boost the confidence in the actual delivered dose, bridging the gap between machine QA and machine settings during dose delivery.^33^, ^34^

## 5 CONCLUSION

Commercially available 3D Monte Carlo IDC software was successfully commissioned and validated. Good agreement was observed between the dose calculation algorithms provided by the Multiplan TPS and SciMoCa based on reference measurements in water. The use of the IDC in clinical practice has been validated by analyzing a set of 84 patient plans using SPC and a comparison to pre‐treatment measurements. After a risk assessment of our QA program, independent dose calculations using SciMoCa have replaced regular patient‐specific QA measurements for the CyberKnife in our institute.

## CONFLICT OF INTEREST

MA and MS are associated with Scientific RT (SciMoCa). In collaboration, the product for the Cyberknife was developed. However, there has been no influence of Scientific RT on the results described in this paper. There is no commercial interest for MM and MH.
